# Adherence to the Strengthening the Reporting of Observational Studies in Epidemiology Statement in Observational Studies Published in Iranian Medical Journals

**DOI:** 10.18502/ijph.v49i8.3896

**Published:** 2020-08

**Authors:** Soheila SHAGHAGHIAN, Behrooz ASTANEH

**Affiliations:** Department of Medical Journalism, School of Paramedical Sciences, Shiraz University of Medical Sciences, Shiraz, Iran

**Keywords:** Iran, Journal, Observational study, Quality, Strengthening the reporting of observational studies in epidemiology (STROBE)

## Abstract

**Background:**

Although much medical knowledge comes from observational research, such studies are more prone to confounding and bias than others. This study was conducted to evaluate the adherence of the observational studies published in Iranian medical journals to the STROBE (strengthening the reporting of observational studies in epidemiology) statement.

**Methods:**

In this cross-sectional study, we selected 150 articles of Iranian medical journals, using multistage sampling from Aug 2016 to Jun 2017. The reported items of the STROBE statement in the articles was determined and considered as the adherence of the articles to the statement. The adherence of the articles with different characteristics was compared.

**Results:**

The adherence of the articles to the statement varied from 24% to 68% with a mean score of 48%±9%. The lowest mean scores were found in the Result (36%) and Method (49%) sections. The adherence was significantly better in the articles published in the journals indexed in PubMed or Web of Knowledge (ISI) databases (*P*<0.001) and those written by cooperation of the authors from other countries (*P*=0.044).

**Conclusion:**

The evaluated articles in our study had not adequately reported the items recommended by the STROBE statement. This indicates deficiency in key elements for readers to assess the validity and applicability of a study.

## Introduction

To optimally manage patients, health care workers need to find all aspects of diseases by researching. Although randomized clinical trials (RCTs) are the most valuable studies, much of health knowledge comes from observational studies (1). Yet, observational studies are more prone to bias because, in them, patients do not perceive risks by random assignment (2). Therefore, readers will find high-quality observational studies, only if researchers present all processes and results of their studies clearly (3).

Nevertheless, observational studies usually were not reported completely (4). Therefore, a group of methodologists, researchers, and editors released Strengthening the Reporting of Observational Studies in Epidemiology (STROBE) statement in 2007 (5). The statement recommended a clear description of what was designed, conducted, and found in the studies (1).

Up to now, several studies evaluated the adherence of different kinds of observational studies, case-control (6), cross-sectional (7), and cohort (8), to the STROBE statement. The studies published in a specific journal (9) or presented in a congress (10) were also evaluated. However, researchers did not find a good quality in their reporting. Moreover, it was shown that although the quality improved over time, it was not affected by the release of the statement (11).

In recent years, many medical journals have been established in Iran (12); however, the quality of the articles published in the journals is dubitable. Evaluating the adherence of the RCTs published in Iranian medical journals to the CONSORT statement, Sarveravan and others found a very weak adherence (13). However, to the best of our knowledge, no research has evaluated the quality of observational studies published in Iran. Identification of shortcomings of the articles can help to establish movements toward standardized reporting. Therefore, we evaluated the adherence of the observational studies and their subsections to the STROBE statement. Furthermore, the associated factors of the adherence such as the language and publication year of the articles were assessed.

## Materials and Methods

In this cross-sectional study, conducted from Aug 2016 to Jun 2017, we evaluated the articles published in Iranian medical journals from 2015 to 2017. The evaluated articles were the observational studies published in Iranian medical journals ranked as “scientific” by “Iranian Commission for Accreditation of Medical Journals” affiliated to Iranian Ministry of Health and Medical Education. We found the name of 352 scientific medical journals on the webpage of the Iranian Commission in Aug 2016. Of them, 70 journals were indexed in PubMed or Web of Knowledge databases (historically called as Institute for Scientific Information [ISI]). The others consisted of 132 English language and 150 Persian language journals (12).

Considering α=5%, power=80%, and estimated effect size=40%, we calculated the sample size, which was 44 for each group; PubMed/ISI indexed journals, and English language and Persian language non-PubMed/ISI indexed ones. However, we evaluated 150 articles; 50 from each group.

We selected the articles, using multistage sampling. The first stage was stratified random sampling. To do the sampling, we entered the name of all the Iranian medical journals to the SPSS software and randomly selected 10 journals from each group. However, some selected journals were excluded because:

- No observational studies were published in them in 2015, 2016, and 2017.
- Their publication was stopped before 2015.
- The journals were Persian language according to the Iranian Commission list but at the time of our evaluation, their language was English.

To replace the excluded journals, we again randomly selected journals from the list, using SPSS software (Chicago, IL, USA). In the second stage, we selected the latest five observational articles of each selected journal, using convenience sampling. Reading the selected articles, we excluded the articles that their study types were not truly labeled. Data were collected by using researcher-made checklists consisting of two parts. The first part was about descriptive characteristics of the articles including indexing in PubMed/ISI, and their language, publication year and authors’ affiliation. The second part consisted of items evaluating the adherence of the articles to the statement.

Although the STROBE statement has 22 items, there are several subcategories in some of the items. We designed the checklists consisting of all the subcategories. The statement explains about reporting of the three main types of observational studies; therefore, we designed three checklists; 57-item, 56-item, and 53-item checklists for evaluating cohort, case-control, and cross-sectional studies, respectively.

A researcher evaluated all the selected articles, considering the explanation and elaboration of the items (1). However, to calibrate the researcher, 10 articles were evaluated by a team consisting of four faculty members of Shiraz University of Medical Sciences (SUMS). The team members excluded one item, “Describe any efforts to address potential sources of bias”, from the checklists. To enhance the validity of the research, the researcher consulted with epidemiologists and statisticians whenever it was necessary.

To score the conformity of the article to each item of the checklists, the researcher marked the conformed, partially conformed, and non-conformed items as 1, 0.5, and 0, respectively. Moreover, the non-applicable items were considered as missing and to adjust the effect of them, we divided the summation of the scores of each article by the total number of its applicable items. Afterward, the score of each article was calculated based on the total score of 100. Using the same method, each section of the articles was also scored between 0 and 100.

The collected data were analyzed using SPSS software (ver.18, Chicago, IL, USA). The adherence of the articles to the statement was reported by mean (±SD).

To evaluate the effect of partially conforming items, we conducted a sensitivity analysis, using two scoring systems. The partially conforming items were analyzed similar to non-conforming and conforming items, scored 0 and 1, in the first and second scoring system, respectively. The mean scores of the articles in each scoring system were calculated and considered as the worst and the best scores that the article adherence might be taken.

We compared the adherence of the articles in different groups, using independent sample t-test, and one-way analysis of variance (ANOVA) with Tukey’s HSD for multiple comparisons. To control the effect of possible confounding factors, we entered all the article characteristics into a multiple regression model with adherence of the articles to the statement as dependent variable.

The study was approved by the Research Ethics Committee of SUMS. To consider ethical issue, the score of each evaluated journal was confidential.

## Results

**Fig. 1:**
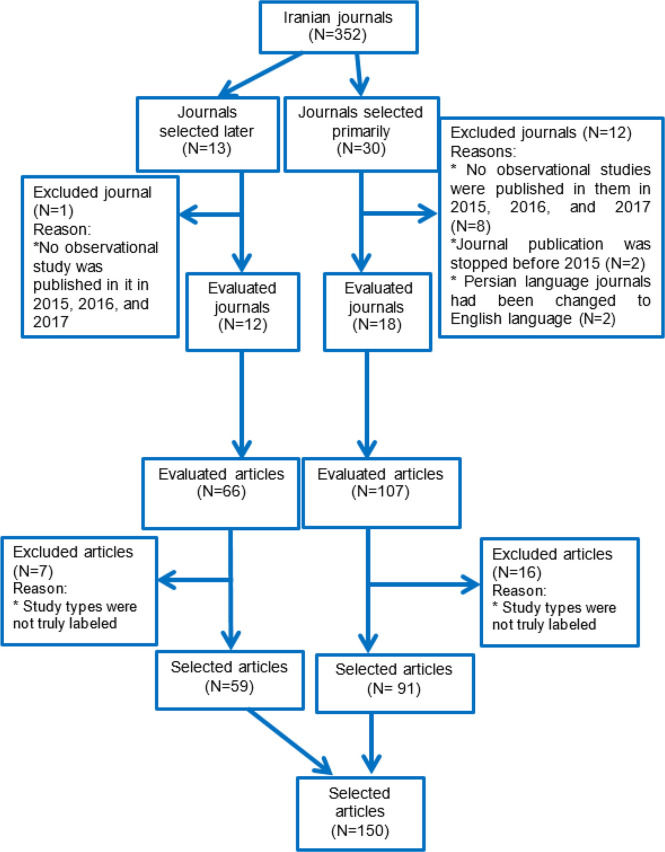
Flow chart shows how the journals and articles were selected

Of the 30 primarily selected journals, 12 were excluded and replaced; five PubMed/ISI indexed, and four English and three Persian-language non-PubMed/ISI indexed ones. To select 150 articles, we evaluated 173 observational articles; 23 were excluded because their study types were not truly labeled (Fig. 1).

Of the evaluated articles, the study design in 124 (82.7%), 21 (14%), and 5 (3.3%) were cross-sectional, case-control, and cohort, respectively. Most of them (70%) were published in 2016 (Table 1).

**Table 1:** Characteristics of the studied articles and their effect on the adherence of the articles to the STROBE statement (N=150)

***Characteristics of the articles***	***N (%)***	***Univariate Analysis***		***Multiple regression analysis***	
		***Adherence (%) (Mean±SD)***	***P-value***	***β***	***P-value***
Language					
-English	100 (66.7)	49±9	0.018^*^	− 0.004	0.834
-Persian	50 (33.3)	45±9		-	-
Indexed in					
-PubMed/ISI	50 (33.3)	52±8	<0.001^*^	0.072	<0.001
-Other databases	100 (66.7)	45±9		-	-
Publication year^¶^					
-2015	13 (8.7)	42±7^a^	0.028^**^	− 0.043	− 0.098
-2016	105 (70.0)	49±9^b^		0.042	0.149
-2017	32 (21.3)	46±10^ab^			
Authors’ affiliation					
-Iran	119 (79.3)	47±9	0.061^*^	-− 0.069	− 0.044
-Iran & other countries	7 (4.7)	54±11			
-No Iran	24 (16.0)	50±9		<0.001	0.966

ISI: Institute of scientific information // N: Number // SD: Standard Deviation

* Independent sample T- test //

** One Way ANOVA

¶ Different letters show statistically significant differences.

The percentage of the articles conforming to each item of the statement is shown in Table 2. Some important points had been reported only by a few articles. Only 20.7%, 5.3%, and 3.3% of the articles had reported sample size calculations, number of the participants with missing data, and the generalizability (external validity) of the study, respectively.

**Table 2:** Adherence of the articles published in Iranian medical journals to the STROBE statement (N=150)

***Article part***	***Items of the STROBE statement checklist***	***Values N (%)***			
		***Conforming***	***Partially conforming***	***Non-conforming***	***Non-applicable***
Title & abstract	Indicate the study’s design in the title or the abstract	88 (58.7)	2 (1.3)	60 (40.0)	0 (0.0)
	An informative and balanced abstract	67 (44.7)	82 (54.7)	1 (0.7)	0 (0.0)
Introduction	Explain the scientific background of the investigation	118 (78.7)	28 (18.7)	4 (2.7)	0 (0.0)
	Explain the rationale for the investigation being reported	92 (61.3)	51 (34.0)	7 (4.7)	0 (0.0)
	State the goal of the study	114 (76.0)	30 (20.0)	6 (4.0)	0 (0.0)
	State specific objectives or pre-specified hypotheses	19 (12.7)	12 (8.0)	112 (74.7)	7 (4.7)
Methods	**Study design:**				
	Present study design early in the paper	92 (61.3)	1 (0.7)	57 (38.0)	0 (0.0)
	**Setting:**				
	Describe the setting and locations of the study	124 (82.7)	8 (5.3)	18 (12.0)	0 (0.0)
	Describe the period of recruitment and data collection	94 (62.7)	3 (2.0)	53 (35.3)	0 (0.0)
	Describe the period of exposure	9 (6.0)	1 (0.7)	6 (4.0)	134 (89.3)
	Describe the period of follow-up	8 (5.3)	0 (0.0)	4 (2.7)	138 (92.0)
	**Participants:**				
	Give the Inclusion criteria of participants	104 (69.3)	13 (8.7)	33 (22.0)	0 (0.0)
	Give the source population of participants	127 (84.7)	11 (7.3)	12 (8.0)	0 (0.0)
	Give methods of selection of participants (sampling)	86 (57.3)	20 (13.3)	44 (29.3)	0 (0.0)
	Give methods of follow-up (only for cohort studies, N=5)	1 (20.0)	0 (0.0)	2 (40.0)	2 (40.0)
	Give the rationale for the choice of cases and controls (for case-control studies, N=21)	3 (14.3)	1 (4.8)	13 (61.9)	4 (19.0)
	For matched studies, give matching criteria (for cohort and case-control studies, N=26)	7 (26.9)	1 (3.8)	2 (7.7)	16 (61.6)
	For matched studies, give number of controls per case (for case-control studies, N=21)	4 (19.0)	0 (0.0)	6 (28.6)	11 (52.4)
	For matched studies, give number of exposed and unexposed (for cohort studies, N=5)	1 (20.0)	0 (0.0)	0 (0.0)	4 (80.0)
	**Variables:**				
	Define all outcomes	124 (82.7)	12 (8.0)	13 (8.7)	1 (0.7)
	Define all exposures	88 (58.7)	20 (13.3)	31 (20.7)	11 (7.3)
	Define all potential confounders	31 (20.7)	9 (6.0)	99 (66.0)	11 (7.3)
	Define all effect modifiers	0 (0.0)	0 (0.0)	143 (95.3)	7 (4.7)
	**Data sources/measurement:**				
	Give methods of measurement	118 (78.7)	17 (11.3)	15 (10.0)	0 (0.0)
	Give sources of data for methods of measurement	86 (57.3)	16 (10.7)	43 (28.7)	5 (3.3)
	Describe comparability of measurement methods between two groups	7 (4.7)	1 (0.7)	28 (18.7)	114 (76.0)
	**Bias:**				
	Describe any efforts to address potential sources of bias	-	-	-	-
	**Study size:**				
	Report of sample size calculations	31 (20.7)	5 (3.3)	113 (75.3)	1 (0.7)
	**Quantitative variables:**				
	Description about groupings chosen for quantitative variables	49 (32.7)	2 (1.3)	29 (19.3)	70 (46.7)
	Description about why the groupings chosen for quantitative variables	24 (16.0)	4 (2.7)	50 (33.3)	72 (48.0)
	**Statistical methods:**				
	Description of unadjusted statistical methods	132 (88.0)	0 (0.0)	9 (6.0)	9 (6.0)
	Description of the statistical methods used to control for confounding	62 (41.3)	0 (0.0)	79 (52.7)	9 (6.0)
	Description of statistical methods used to examine subgroups and interactions	0 (0.0)	0 (0.0)	143 (95.3)	7 (4.7)
	Description of how missing data were addressed	0 (0.0)	0 (0.0)	150 (100.0)	0 (0.0)
	Explanation about how loss to follow-up was addressed (cohort studies, N=5)	0 (0.0)	0 (0.0)	5 (100.0)	0 (0.0)
	Explanation about how matching of cases and controls was addressed (only case-control studies, N=21)	0 (0.0)	0 (0.0)	11 (52.4)	10 (47.6)
	Describe analytical methods taking account of sampling strategy (cross sectional studies, N=124)	0 (0.0)	0 (0.0)	123 (99.2)	1 (0.8)
	Description about any sensitivity analyses	1 (0.7)	0 (0.0)	148 (98.7)	1 (0.7)
Results	**Participants:**				
	Report numbers of individuals eligible for the study	10 (6.7)	1 (0.7)	139 (92.7)	0 (0.0)
	Report numbers of individuals included in the study	89 (59.3)	0 (0.0)	61 (40.7)	0 (0.0)
	Give reasons for non-participation at each stage	4 (2.7)	5 (3.3)	140 (93.3)	1 (0.7)
	Use of a flow diagram for showing participants	0 (0.0)	0 (0.0)	149 (99.3)	1 (0.7)
	**Descriptive data:**				
	Give characteristics of study participants	103 (68.7)	19 (12.7)	28 (18.7)	0 (0.0)
	Give information on exposures	105 (70.0)	16 (10.7)	19 (12.7)	10 (6.7)
	Give information on potential confounders	41 (27.3)	7 (4.7)	93 (62.0)	9 (6.0)
	Indicate number of participants with missing data for each variable of interest	8 (5.3)	1 (0.7)	141 (94.0)	0 (0.0)
	Summarize follow-up time (only for cohort studies, N=5)	3 (60.0)	0 (0.0)	2 (40.0)	0 (0.0)
	**Outcome data:**				
	Report numbers of outcome events or summary measures over time (only cohort studies, N=5)	5 (100.0)	0 (0.0)	0 (0.0)	0 (0.0)
	Report numbers in each exposure category (only case-control studies, N=21)	18 (85.7)	2 (9.5)	1 (4.8)	0 (0.0)
	Report numbers of outcome events or summary measures (only cross sectional studies, N=124)	109 (87.9)	2 (1.6)	12 (9.7)	1 (0.8)
	**Main results:**				
	Give unadjusted estimates and their precision	125 (83.3)	13 (8.7)	1 (0.7)	11 (7.3)
	Give confounder-adjusted estimates and their precision	56 (37.3)	3 (2.0)	82 (54.7)	9 (6.0)
	Make clear which confounders were adjusted for	48 (32.0)	2 (1.3)	91 (60.7)	9 (6.0)
	Make clear why the confounders were included	5 (3.3)	0 (0.0)	136 (90.7)	9 (6.0)
	Report category boundaries when continuous variables were categorized	46 (30.7)	9 (6.0)	26 (17.3)	69(46.0)
	Consider translating estimates of relative risk into absolute risk	0 (0.0)	0 (0.0)	0 (0.0)	150 (100)
	**Other analyses:**				
	Report analyses of subgroups and interaction	1 (0.7)	1 (0.7)	141 (94.0)	7 (4.7)
	Report sensitivity analyses	2 (1.3)	0 (0.0)	148 (98.7)	0 (0.0)
Discussion	**Key results:**				
	Summarize key results in the beginning of discussion	66 (44.0)	23 (15.3)	61 (40.7)	0 (0.0)
	**Limitations:**				
	Discuss limitations of the study	81 (54.0)	9 (6.0)	60 (40.0)	0 (0.0)
	**Interpretation:**				
	Give interpretation of results considering results from similar studies, and other relevant evidence	132 (88.0)	13 (8.7)	5 (3.3)	0 (0.0)
	**Generalizability:**				
	Discuss the generalizability (external validity) of the study	5 (3.3)	0 (0.0)	145 (96.7)	0 (0.0)
Other information	**Funding:**				
	Give the source of funding	81 (54.0)	7 (4.7)	62 (41.3)	0 (0.0)

Of some items, although some subcategories had been mentioned in acceptable proportion of the articles, other subcategories had been reported by a few ones. While 32.7% of the articles had described “groupings chosen for quantitative variables”, only 16.0% had described about “why the groupings were chosen for them”. Although the goal of study had been stated in 76.0%, the specific objectives had been stated only in 12.7%.

Similarly, 88.0% and 41.3% of the articles had described the used unadjusted, and confounder adjusted statistical analysis in the Method, respectively. However, no articles had reported the statistical methods used to examine subgroups and interactions; to control the effect of sampling strategy; and to describe how missing data, loss to follow-up, and matching of cases and controls were addressed. Furthermore, only one article had described sensitivity analysis. Likewise, in result section, 83.3%, and 37.3% had reported unadjusted and confounder-adjusted analysis but only 3.3% had made clear why the confounders were included. In addition, only 1, and 2 studies had reported analyses of subgroups and interaction, and sensitivity analysis, respectively (Table 2).

**Fig. 2:**
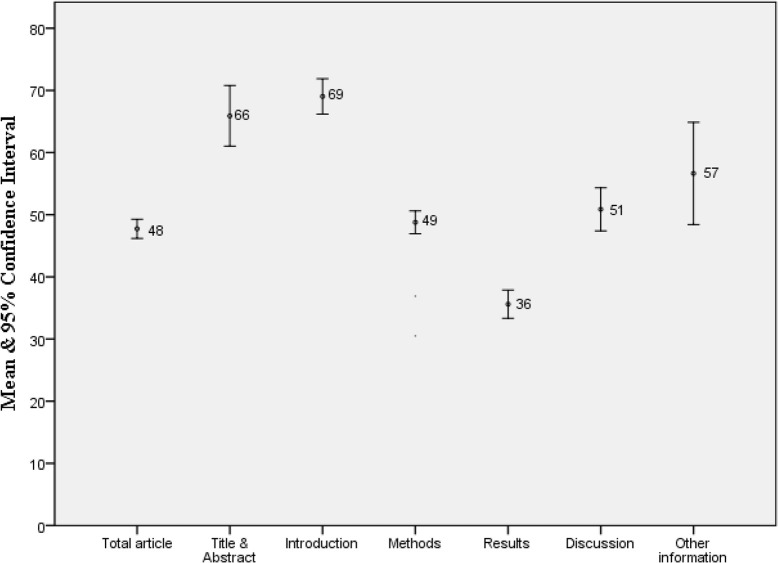
The adherence of sections of articles published in Iranian Medical journals to the STROBE statement

The adherence score of each evaluated article varied from 24% to 68% (Mean±SD: 48%±9%). Comparing the means of the article sections, we found the least and the highest score in Result and Introduction, respectively (Fig. 2).

The means of the articles calculated in the sensitivity analysis were very close to the calculated mean. The best and worst means were 45%±10% and 51%±9%, respectively.

In univariate analysis, the adherence of the articles was significantly associated with their language (*P*=0.018) and publication year (*P*=0.028), and whether they indexed in PubMed/ISI or not (*P*<0.001). In multiple regression analysis, the adherence of the articles published in PubMed/ISI indexed journals was significantly better than the others (*P*<0.001). In contrast, the adherence was not significantly associated with their language and publication year. Furthermore, the articles whose authors were affiliated with two or several countries including Iran had more adherence than the ones whose authors’ affiliation was only Iran (*P*=0.044, Table 1).

## Discussion

This study assessed the adherence of the observational studies published in Iranian medical journals to the STROBE statement and the factors associated with the adherence. Our result showed a large proportion of unreported STROBE items in the articles. Furthermore, the adherence was significantly better in multicenter studies and in the articles published in PubMed/ISI indexed journals. The evaluated articles in our study, similar to the ones in other studies (2, 6, 14, 15) had reported averagely half of the items recommended by the statement. This highlighted a clear need to improve the quality of the reporting.

Although Method and Result are the most important parts of articles, our study, similar to another study (7), showed the least adherence in the sections. Unclear presentation of what was done and found can lead to difficult interpretation of studies and should be prevented.

Sample size is the most important factor to determine the statistical power of a research. Nevertheless, our results, similar to other studies (3, 15–17) showed poor reporting of sample size calculations. Therefore, authors and scientists should be trained to report not only the number of participants but also the process of calculating the number.

Missing data can influence the generalizability of the study or cause biases. Yet, in the evaluated articles in our study and other studies (3, 15–18) the participants excluded from research had rarely been reported. Furthermore, there was not a significant improvement in the reporting of this item after establishment of the STROBE (16).

According to the STROBE, investigators should explain not only “which groupings were chosen for quantitative variables” but also “why the groupings were chosen”.^1^ However, in our study, only a few number of the articles had described the reason. Because the articles published after establishment of the statement significantly reported the item better (16), adequate introduction of the statement can improve its reporting.

Objectives are the detailed aims of the study and should be stated in Introduction (1). Nevertheless, in our study, similar to other studies (3, 16), objectives were stated only in a few ones although the goal of the study was stated in much of the articles. Statistical analysis should be clearly described in Method and reported in Result. If both unadjusted and confounder-adjusted analyses are reported, readers will be able to judge by how much, and in what direction, potential confounders change effect estimate (2). In addition, the reason that the confounders were included in the adjusted analyses is so important because defining associations between various data depend not only on the data but also on the design of the study. Furthermore, the analyses of subgroups and interactions and sensitivity analyses should be reported to display potential interaction between risk factors, and to estimate the probable range of variation in outcome. However, in our study, similar to other studies (3, 7, 15, 17, 19), only few articles reported the analyses.

In our study, the articles that their authors were from two or several countries including Iran were more conformed to the statement than those whose authors were only from Iran. The result highlighted the importance of designing large and multicenter studies with contribution of the authors from different countries.

Our study, similar to other studies (20), showed that the articles published in PubMed/ISI indexed journals were significantly more conformed to the statement. Yet, in our study and other studies (19, 20), the conformity was not satisfactory even in the articles published in PubMed/ISI indexed journals. Therefore, improving the quality of all articles, including those published in PubMed/ISI indexed journals is necessary.

To improve the quality of observational studies, we suggest the following recommendations:

- Increasing the awareness of researchers and editors about the importance of reporting articles compatible with the STROBE
- Establishing workshops for training of researchers to write their articles compatible to the statement
- Endorsing the statement in the instructions for authors of journals
- Requiring authors to submit a checklist with sufficient text excerpted from the manuscript to explain how they accomplished all applicable items of the statement
- Considering the statement in the review process of articles

Despite our great efforts to conduct a well-designed study, this study had some limitations. The most important was the subjective nature of the scoring. To decrease the extent of the problem, the evaluation of all articles was conducted by one of the researchers, a medical doctor expert in epidemiology and statistics.

The second limitation was about the scoring of the partially conformed items. While all the items received the same score, they had different degrees of conformation. To show the extent of such problem, we conducted a sensitivity analysis. However, the best and worst means in the analysis were very close to the previously calculated mean. Therefore, the effect of the items was ignorable. Another limitation was the same scoring of all the items of the statement while they did not have the same weight in terms of their effect on the validity of articles. The next limitation occurred because it was impossible to blind the researcher evaluating the articles to the name of authors or journals. However, the researcher did not have any competing interest to the evaluated articles. Furthermore, because the evaluated articles were not graded in one sitting, the effects of grading variability or grader fatigue might occur.

## Conclusion

Compliance with the STROBE statement substantially increases the quality of reporting observational studies. However, our study shows low compliance of the observational studies published in Iranian Medical journals to the statement in many items, especially those related to Result and Method. Interventional programs should be established to improve the situation.

## Ethical considerations

Ethical issues (Including plagiarism, informed consent, misconduct, data fabrication and/or falsification, double publication and/or submission, redundancy, etc.) have been completely observed by the authors.
